# A structural equation model for imaging genetics using spatial transcriptomics

**DOI:** 10.1186/s40708-018-0091-0

**Published:** 2018-11-02

**Authors:** Sjoerd M. H. Huisman, Ahmed Mahfouz, Nematollah K. Batmanghelich, Boudewijn P. F. Lelieveldt, Marcel J. T. Reinders, for the Alzheimer’s Disease Neuroimaging Initiative

**Affiliations:** 1 Delft Bioinformatics Lab, Delft University of Technology, Delft, The Netherlands; 2 Leiden Computational Biology Center, Leiden University Medical Center, Leiden, The Netherlands; 3 Division of Image Processing, Department of Radiology, Leiden University Medical Center, Leiden, The Netherlands; 4 Department of Biomedical Informatics, University of Pittsburgh, Pittsburgh, PA USA

**Keywords:** Imaging genetics, Brain genetics, Structural equation modelling, ADNI, Allen Brain Atlas

## Abstract

Imaging genetics deals with relationships between genetic variation and imaging variables, often in a disease context. The complex relationships between brain volumes and genetic variants have been explored with both dimension reduction methods and model-based approaches. However, these models usually do not make use of the extensive knowledge of the spatio-anatomical patterns of gene activity. We present a method for integrating genetic markers (single nucleotide polymorphisms) and imaging features, which is based on a causal model and, at the same time, uses the power of dimension reduction. We use structural equation models to find latent variables that explain brain volume changes in a disease context, and which are in turn affected by genetic variants. We make use of publicly available spatial transcriptome data from the Allen Human Brain Atlas to specify the model structure, which reduces noise and improves interpretability. The model is tested in a simulation setting and applied on a case study of the Alzheimer’s Disease Neuroimaging Initiative.

**Electronic supplementary material:**

The online version of this article (10.1186/s40708-018-0091-0) contains supplementary material, which is available to authorized users.

## Introduction

The aim of imaging genetics studies is to find associations between genetic variants and imaging features, often in a disease context [1]. This scheme extends beyond traditional genome-wide association studies (GWAS) by identifying genetic associations of imaging biomarkers with the assumption that these biomarkers are a more direct reflection of the genetic effects. Thus, they could provide a stronger association signal [2]. Additionally, the identified associations are likely to provide new insights into the underlying disease mechanisms as well as new hypotheses about the anatomical and/or functional locations involved in complex diseases [3].

So far, imaging genetics studies have been largely focused on the brain [1, 3–6], despite efforts to extend their application to other fields [7]. Several large consortia have gathered data from thousands of subjects to understand the effects of genetic variants on brain structure and function [8]. One of the hallmark sources for imaging genetics studies is the Alzheimer’s Disease Neuroimaging Initiative (ADNI) database [9]. This database contains single nucleotide polymorphism (SNP) and structural MRI data for Alzheimer’s patients, individuals with late mild cognitive impairment, and cognitive normal controls.

One of the largest challenges facing imaging genetics studies is the statistical power needed to identify reliable associations. In a typical GWAS, researchers have to correct for the number of independent tests performed (i.e. number of independent SNPs tested) in order to limit the number of false-positive discoveries. However, a genome-wide brain-wide imaging genetic study will not only have to correct for the number of independent SNPs, but also for the number of independent imaging features tested. As a result, many studies are underpowered to identify reliable associations. One of the largest imaging genetics studies [10] analysed over 30,000 individuals within the Enhancing Neuro Imaging Genetics through Meta-Analysis (ENIGMA) consortium. They performed a genome-wide association of SNPs with seven brain volumes and identified only eight genome-wide significant SNPs.

Despite the high dimensionality of the imaging data (millions of voxels), the actual number of independent tests for which we need to correct in an imaging genetics study is far smaller than the number of voxels. Due to the spatial relationships between voxels, measurements from neighbouring voxels are usually highly correlated. A common approach is to test genetic associations for anatomically defined brain regions [2]. Several studies have shown that both neuroanatomical parcellation and connectivity of the brain are strongly reflected in gene expression patterns across the brain [11–13]. The public availability of brain transcriptome atlases from the Allen Institute for Brain Science [14] provides an opportunity to use these transcriptional signatures to group the anatomically defined brain regions, further limiting the number of effective tests.

Several methods have been proposed to identify associations between genetic variants and imaging features by applying dimension reduction, such as variations of canonical correlation analysis [15], and independent component analysis (commonly used in a functional MRI context) [4]. Others have opted to model the interactions between the different data types explicitly, for instance using graphical Bayesian models [16, 17] which capture a more mechanistic causal view of the data. These models consist of a directed acyclic graph, which can easily be made to incorporate covariates, including possible confounding factors. Both of these studies use relatively small candidate SNP sets, because they aim for understanding SNP–brain relationships rather than the discovery of genome-wide associations. However, these Bayesian models are quite challenging to specify and fit.

In this work, we propose a method to identify associations between candidate genetic variants and imaging features allowing for the incorporation of prior knowledge. The proposed method combines a graphical model with dimension reduction to model the effect of SNPs on brain imaging features through a set of latent variables. We use a maximum likelihood structural equation modelling (SEM) approach to find the edge weights of our model [18]. By performing dimensionality reduction within the model, we reduce the number of parameters to be estimated. In addition, the model allows for easy incorporation of information from the Allen Human Brain Atlas [12] to inform the grouping of brain regions based on the similarity of their transcriptional profiles.

Our model uses the transcriptional profiles for grouping because we consider gene expression to be an intermediate phenotype, that links SNPs to brain imaging features. Most disease-associated SNPs are located near regulatory regions of the genome [19], and the effects of SNPs on expression tend to be tissue and cell type specific [20]. Gene expression data of brain regions reflect cell type composition and anatomical similarity [12] and capture a wide range of brain-specific molecular pathways [21]. For these reasons the region groups in the dimension reduction are based on spatial gene expression data of the brain.

## Materials and methods

The interplay between genetic variation, brain anatomy, and disease symptoms is complex. We use a structural equation model with latent variables [18] to model these relationships. We pose that the genetic variation is exogenous; in other words, the genetic variation in a study population is not caused by disease or brain anatomy. This variation does have an effect on the brain. For example, in Alzheimer’s disease, genetic variants may influence the immune response and amyloid $\beta$ concentrations in the brain, which may in turn lead to shrinkage in several brain areas [22]. Large-scale imaging initiatives, such as ADNI, offer a possibility to study this shrinkage of brain regions. This can be estimated from MRI data of diseased individuals and controls, and expressed in cortical thickness and subcortical volume measurements.

In our graphical model, we define groups of brain regions, based on the transcriptional profiles of these areas in the healthy brain. Areas that share patterns of gene expression in a normal brain may be similarly affected by genetic variations. For each of the region groups, we introduce one latent variable. This latent variable is affected by the genetic variations and causes changes in relevant brain regions. This makes our model similar to principal component analysis (PCA) on sets of brain regions, combined with a regression for the latent variables. However, in our model the weights are estimated together, and the latent variables reflect not only the correlations between the regions (as in a conventional PCA), but also those between regions and SNPs and among the SNPs.

### Variables used

We model the relationship between single nucleotide polymorphisms (SNPs) and brain region measurements. Let ${\mathbf {g}}_i \in {\mathbb {R}}^{p}$ be a vector of centred (zero-mean) SNP values, and ${\mathbf {x}}_i \in {\mathbb {R}}^{q}$ a vector of centred (zero-mean) and scaled (${\text {sd}}=1$) brain region measurements, both for individual *i*. The reason both types of measurements are centred is to eliminate intercepts from the model. The brain measurements are, in addition, scaled to unit variance to compensate for the considerably larger variance in thickness or volume for larger brain areas. The genetic variants and brain measurements are connected in the model by a set of latent variables, ${\mathbf {z}}_i \in {\mathbb {R}}^{m}$.

In addition to the variables included in the model, we have two other sources of information. In defining the model structure, we make use of external information on the brain region measurements, in the form of brain region groups with a shared transcriptional profile. These groups are defined based on the spatial gene expression data of the healthy adult brain. Finally, the goal is to understand disease-related phenotypes. The disease labels are not used in the modelling stage. However, we hypothesize that if the variation in the data is related to a disease state, the latent variables will reflect this. After model fitting, we therefore associate each individual’s estimated latent variable score with his or her disease status.

### The graphical model

We model the relationship between brain SNP values and brain region measurements in a structural equation model (SEM). It consists of two parts. The first part is a linear model for brain region measurements as a function of the latent variables,

1 $$\begin{aligned} {\mathbf {x}}_i = {\mathbf {B}}{\mathbf {z}}_i+\varvec{\zeta}_{i}, \end{aligned}$$

where ${\mathbf {x}}_i$ contains the observed brain region measurements, ${\mathbf {z}}_i$ are the latent variables, and $\varvec{\zeta}_{i}$ is a zero-mean normally distributed error variable. The matrix ${\mathbf {B}}$ contains the weights of the latent variables that explain the brain region measurements. The second part of the SEM is a linear model for these latent variables as a function of the SNP values,

2 $$\begin{aligned} {\mathbf {z}}_i = {\mathbf {A}}{\mathbf {g}}_i+\varvec{\varepsilon }_i, \end{aligned}$$

where ${\mathbf {g}}_i$ contains the observed SNP measurements and $\varvec{\varepsilon }_i$ is a zero-mean normally distributed error variable. The matrix ${\mathbf {A}}$ contains regression weights, representing the effects of the SNPs on the latent variables. Combined, these equations mean that region changes are viewed as a manifestation of the latent values, while the SNP values are considered causal to them. The latent variables represent some intermediate phenotype, related to the molecular state of the connected brain regions.

**Fig. 1:**
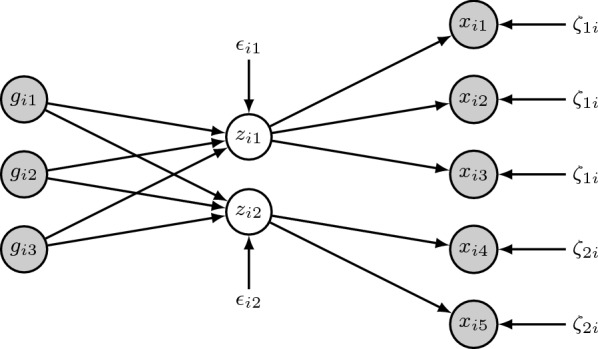
The graphical structural equation model. Observed variables are shown in grey circles, latent variables in white circles, and error variables without circles. This example contains two latent variables, both with their own set of observed brain region measurements. This structure, where the latent variables define groups of region measurements, is defined by prior knowledge on these brain regions. We use spatially resolved gene expression data of the healthy human brain to define these region groups

The number of latent variables is equal to the number of brain region groups, which are defined based on external spatial gene expression data. A region group contains the brain regions with a similar transcriptional profile, as these may react similarly to differences in genetic background. We restrict each latent variable to only predict the brain region measurements for its own region group. This results in a restriction on the weight matrix ${\mathbf {B}}$, where each latent variable (corresponding to a column in ${\mathbf {B}}$) has a unique set of nonzero entries. Figure 1 shows the model for two latent variables, where we can see that each latent variable is connected to its own set of brain regions.

### Model implied covariance

In linear Gaussian structural equation modelling, we learn the parameters of a model by optimizing the correspondence between the observed covariance ${\mathbf {S}}$ (from the data) and the model implied covariance $\varSigma$. The model implied covariance can be divided into a block matrix, by defining

$$\varSigma = \left[\begin{array}{cc} \varSigma _{gg}&\varSigma _{gx} \\ \varSigma _{xg}&\varSigma _{xx} \end{array}\right].$$

Note that this implied covariance does not contain any components for the latent variables in ${\mathbf {z}}$. The latent variables are not observed, and therefore, we cannot use their observed covariance in fitting the model.

The elements of the implied covariance can be parameterized in terms of the model coefficients. The first element is

$$\begin{aligned} \varSigma _{gg} = {\mathbf {E}}\left[ {\mathbf {g}}{\mathbf {g}}^{\mathrm{T}}\right] . \end{aligned}$$

This is the covariance of the SNPs (since these values are centred). The SNPs are exogenous in our model: ${\mathbf {g}}$ does not have any causal variables within our model. As a result, the implied covariance of the SNPs is not parameterized in terms of model coefficients. We can estimate this covariance term simply by taking the observed covariance between the SNPs.

The next element of the implied covariance matrix is

$$\begin{aligned} \varSigma _{xg}&= {\mathbf {E}}\left[ {\mathbf {x}}{\mathbf {g}}^{\mathrm{T}}\right] \\&= {\mathbf {E}}\left[ {\mathbf {B}}{\mathbf {A}}{\mathbf {g}}{\mathbf {g}}^{\mathrm{T}}+\varvec{\varepsilon }{\mathbf {g}}^{\mathrm{T}}+\varvec{\zeta }{\mathbf {g}}^{\mathrm{T}}\right] , \end{aligned}$$

and similarly

$$\begin{aligned} \varSigma _{gx}&= {\mathbf {E}}\left[ ({\mathbf {x}}{\mathbf {g}}^{\mathrm{T}})^{\mathrm{T}}\right] \\&= {\mathbf {E}}\left[ {\mathbf {g}}{\mathbf {g}}^{\mathrm{T}}{\mathbf {A}}^{\mathrm{T}}{\mathbf {B}}^{\mathrm{T}}+{\mathbf {g}}\varvec{\varepsilon }^{\mathrm{T}}+{\mathbf {g}}\varvec{\zeta }^{\mathrm{T}}\right] . \end{aligned}$$

The final element of the implied covariance matrix is the model implied covariance among the brain regions. This is given by

$$\begin{aligned} \varSigma _{xx}&= {\mathbf {E}}\left[ {\mathbf {x}}{\mathbf {x}}^{\mathrm{T}}\right] \\&= {\mathbf {E}}\left[ {\mathbf {B}}{\mathbf {A}}{\mathbf {g}}{\mathbf {g}}^{\mathrm{T}}{\mathbf {A}}^{\mathrm{T}}{\mathbf {B}}^{\mathrm{T}} +{\mathbf {B}}{\mathbf {A}}{\mathbf {g}}\varvec{\varepsilon }^{\mathrm{T}}{\mathbf {B}}^{\mathrm{T}} +{\mathbf {B}}\mathbf {A}{\mathbf {g}}\varvec{\zeta }^{\mathrm{T}} \right. \\&\left. \quad +\,{\mathbf {B}}\varvec{\varepsilon }{\mathbf {g}}^{\mathrm{T}}{\mathbf {A}}^{\mathrm{T}}{\mathbf {B}}^{\mathrm{T}} +{\mathbf {B}}\varvec{\varepsilon }\varvec{\varepsilon }^{\mathrm{T}}{\mathbf {B}}^{\mathrm{T}} +{\mathbf {B}}\varvec{\varepsilon }\varvec{\zeta }^{\mathrm{T}}\right. \\&\left. \quad +\,\varvec{\zeta }{\mathbf {g}}^{\mathrm{T}}{\mathbf {A}}^{\mathrm{T}}{\mathbf {B}}^{\mathrm{T}} +\varvec{\zeta }\varvec{\varepsilon }^{\mathrm{T}}{\mathbf {B}}^{\mathrm{T}} +\varvec{\zeta }\varvec{\zeta }^{\mathrm{T}}\right] . \end{aligned}$$

### Model assumptions and estimation

Some elements of the implied covariance are often assumed to be zero. These assumptions lead to a strong simplification of the implied covariance. It is common in a regression setting to pose that the predictor variables and error variables are independent. In our case, the error independence assumption leads to ${\mathbf {g}}\varvec{\varepsilon }^{\mathrm{T}} = \varvec{\varepsilon }{\mathbf {g}}^{\mathrm{T}} = 0$. In addition, we assume that the errors in the brain region predictions (Eq. 1) are independent of the errors in the latent variable predictions (Eq. 2). This means that $\varvec{\zeta }\varvec{\varepsilon }^{\mathrm{T}} = \varvec{\varepsilon }\varvec{\zeta }^{\mathrm{T}} = 0$. Finally, we assume that the errors in brain region prediction are independent of the SNPs, so ${\mathbf {g}}\varvec{\zeta }^{\mathrm{T}} = \varvec{\zeta }{\mathbf {g}}^{\mathrm{T}} = 0$.

As a result of these assumptions, the full implied covariance matrix of the model reduces to

3 $$\begin{aligned}&\left[\begin{array}{cc} \varSigma _{gg}&\quad \varSigma _{gx} \\ \varSigma _{xg}&\quad \varSigma _{xx} \end{array}\right]\nonumber \\&\quad = {\mathbf {E}} \left[\begin{array}{cc} {\mathbf {g}}{\mathbf {g}}^{\mathrm{T}}&\quad {\mathbf {g}}{\mathbf {g}}^{\mathrm{T}}{\mathbf {A}}^{\mathrm{T}}{\mathbf {B}}^{\mathrm{T}} \\ {\mathbf {B}}{\mathbf {A}}{\mathbf {g}}{\mathbf {g}}^{\mathrm{T}}&\quad {\mathbf {B}}{\mathbf {A}}{\mathbf {g}}{\mathbf {g}}^{\mathrm{T}}{\mathbf {A}}^{\mathrm{T}}{\mathbf {B}}^{\mathrm{T}}+{\mathbf {B}}\varvec{\varepsilon }\varvec{\varepsilon }^{\mathrm{T}}{\mathbf {B}}^{\mathrm{T}} +\varvec{\zeta }\varvec{\zeta }^{\mathrm{T}} \end{array}\right]. \end{aligned}$$

For normally distributed data, the maximum likelihood estimate of the covariance matrix is

4 $$\begin{aligned} \max _\varSigma \left( -\log (|\varSigma |)-{\text {tr}}\left( {\mathbf {S}}\varSigma ^{-1}\right) \right) , \end{aligned}$$

where ${\mathbf {S}}$ is the observed covariance matrix. The SNP data we use are discrete and can therefore not be considered normally distributed. To compensate for this, we will estimate robust standard errors. In Eq. (4), the covariance $\varSigma$ is parameterized according to Eq. (3), so we can perform the optimization over the parameter values.

Model fitting is performed in the *lavaan* package in *R* [23]. For identifiability, we fix the loading of the first brain region measurement per region group (latent variable) to 1. This does not only fix the scales of the latent variables, but it also has the advantage that the resulting latent variables will have the same direction of effect as the first brain region measurement. For example, a reduction in volume of the first brain region will result in a reduction in the corresponding latent variable. All the error variances on the brain region measurements (variance of $\varvec{\zeta }$) are assumed to be equal within each region group, which is the same as in principal component analysis.

The model fit in *lavaan* yields estimates for ${\mathbf {B}}$, $\mathbf {A}$, and the covariance matrices of the error variables $\varvec{\varepsilon }$ and $\varvec{\zeta }$. Each of these parameter estimates is provided with robust *p* values (for the hypothesis of being equal to zero), when using the *MLM* estimation procedure [23]. Using the estimated model parameters, one can then calculate unbiased Bartlett scores for the latent variables [24].

### Data

*Simulated data* The model is evaluated on both simulated and real data. In the simulation, we first generated SNP values (${\mathbf {g}}_i$) in accordance with Hardy–Weinberg equilibrium. The minor allele frequencies were independently drawn from a beta distribution with shape parameters $\alpha =1$ and $\beta =2$. Then we simulated latent variables (${\mathbf {z}}_i$) as a linear combination of the SNP values, with Gaussian noise (${\hbox {sd}} = 2$). Each of these latent variables determined the region measurements (${\mathbf {x}}_i$) of a set of regions (a region group), with added Gaussian noise (${\hbox {sd}} = 2$). This part of the simulation is in line with Eqs. (1) and (2) and Fig. 1. Finally, we used a logistic model in which a linear combination of some of the latent variables determined the probability of observing a phenotype. These binary phenotypes (disease versus healthy) were then drawn from a Bernoulli distribution.

We simulated 100 independent data sets for 500 individuals. Each time, we set the number of SNPs to 20 and the number of latent variables (and therefore region groups) to 5. We randomly selected 10 SNP-to-latent weights (${\mathbf {A}}$) to be either 1 or $-\,1$. The 5 region groups contain 20, 10, 10, 5, and 5 regions, respectively, for a total of 50 brain region measurements. Each latent variable has latent-to-brain-region weights (in ${\mathbf {B}}$) which were uniformly sampled between 0.5 and 1.5. All other elements of ${\mathbf {B}}$ were set to zero, which effectively restricts each latent variable to affect only its own region group. Finally, two out of the five latent variables were randomly selected to affect the disease probability, with weights of either 10 or $-10$. All other latent-to-phenotype weights were set to zero.

To test the robustness of our method, we also simulated data for a range of alternative parameter settings. We varied the amount of noise in the latent variables (${\mathbf {z}}_i$) and the region measurements (${\mathbf {x}}_i$) between 1 and 5. The number of nonzero SNP-to-latent weights (in ${\mathbf {A}}$) was varied from 2 to 20. Finally, we constructed data sets with misspecified latent-to-brain-region weights (in ${\mathbf {B}}$). To this end, we swapped links between latent variables and regions. In each swap, a region was disconnected from its original latent variable and instead connected to another latent variable. To retain the sizes of the region groups, another region of that second latent variable was then connected to the first latent variable. Each swap therefore resulted in two misspecified links. We made sure not to swap regions back to their original latent variables.

*ADNI data and preprocessing* The real data used in the preparation of this article were obtained from the Alzheimer’s Disease Neuroimaging Initiative (ADNI) database (adni.loni.usc.edu) [9]. The ADNI was launched in 2003 as a public–private partnership, led by Principal Investigator Michael W. Weiner, MD. The primary goal of ADNI has been to test whether serial magnetic resonance imaging (MRI), positron emission tomography (PET), other biological markers, and clinical and neuropsychological assessment can be combined to measure the progression of mild cognitive impairment (MCI) and early Alzheimer’s disease (AD). For up-to-date information, see www.adni-info.org.

The ADNI database contains measurements on a large number of cognitive normal (CN) controls, individuals with late mild cognitive impairment (LMCI), and individuals with Alzheimer’s disease (AD). The measurements in the database include patient demographics, raw and processed MRI data, biomarker data, and SNP data. For the brain volumes we made use of the UCSF cross-sectional FreeSurfer (version 4.3) cortical thickness and white matter parcellation measurements. For the SNPs we made use of the ADNI 1 Illumina Human 610-Quad BeadChip data, with imputation as previously described [17]. In the end, we selected volumes, SNPs, and diagnoses for 746 individuals. These data were split into two equal parts of 373 individuals, one as a training set and one as a validation set, to prevent over-fitting in the modelling process.

Our methodology is not suited to genome-wide analysis. Instead, it tries to find the effects of specific SNPs on a set of latent variables. As candidate SNPs we selected a set of 35 polymorphisms associated with Alzheimer’s disease according to the International Genomics of Alzheimer’s Project (IGAP) study results [25]. IGAP is a two-stage GWAS on individuals of European ancestry for Alzheimer’s disease. In stage 1, IGAP used genotyped and imputed data on 7,055,881 SNPs of 17,008 Alzheimer’s disease cases and 37,154 controls. In stage 2, 11,632 SNPs were genotyped and tested for association in an independent set of 8,572 Alzheimer’s disease cases and 11,312 controls. Finally, a meta-analysis was performed combining results from stages 1 and 2. We selected the known SNPs, stage 1 discoveries, and stage 1 and stage 2 discoveries from table 2, and the suggestive SNPs from supplemental table 4 of [25].

The volume data were present for 112 regions. We corrected it for individual age, gender, and whole brain volume (using linear regression), with the goal of maintaining all meaningful variation in brain region volumes, possibly related to the disease phenotype. For our latent variable model, the brain region volumes were linked to region groups. We defined these region groups based on the transcriptional profiles in the healthy adult human brain, as provided by the Allen Atlas [12]. This gene expression resource contains anatomically labelled measurements taken from six human brains. Regions with measurements in each of the six brains were selected, and the expression values were averaged to obtain a single value for each of the 19,992 genes in each of the 105 regions of the Allen Atlas [21]. We then performed a t-distributed neighbourhood embedding (t-SNE) analysis to obtain a two-dimensional map of the brain regions. Brain regions are placed nearby in this map if they have a similar expression profile across all genes. This map was then used to manually define nine groups of brain regions, as is shown in figure 2 of [21]. The regions of the ADNI data were manually linked to the nine region groups, as shown in Additional file 1: Table S1. The anatomical atlas used for the Allen Atlas is hierarchical: it has a tree-like structure with large regions containing smaller regions. Table 1 shows a higher-level description of the regions in the nine region groups. In most cases the Allen Atlas regions were more general (larger) than the FreeSurfer regions of the ADNI data. Out of the 112 regions, 105 regions were linked to a region group, while the other 7 regions did not have corresponding samples in the Allen Atlas data and were therefore left out.

**Table 1:** The nine region groups (corresponding to the latent variables), with the brain regions they contain

Region group code	ABA region
CrCortex	Cingulate gyrus
CrCortex	Frontal lobe
CrCortex	Insula
CrCortex	Middle frontal gyrus
CrCortex	Occipital lobe
CrCortex	Parahippocampal gyrus
CrCortex	Parietal lobe
CrCortex	Temporal lobe
Hippocam	Hippocampal formation
Amygdala	Amygdala
Striatum	Striatum
DorsThal	Dorsal thalamus
SubCort1	Myelencephalon
SubCort2	Globus pallidus
SubCort2	White matter
ClCortex	Cerebellar cortex
SulcSpac	Sulci and spaces

These are higher-level labels of the Allen Atlas [12]. A full subdivision of the ADNI FreeSurfer regions into these region groups is provided in Additional file 1: Table S1

## Results

### Simulation

**Fig. 2:**
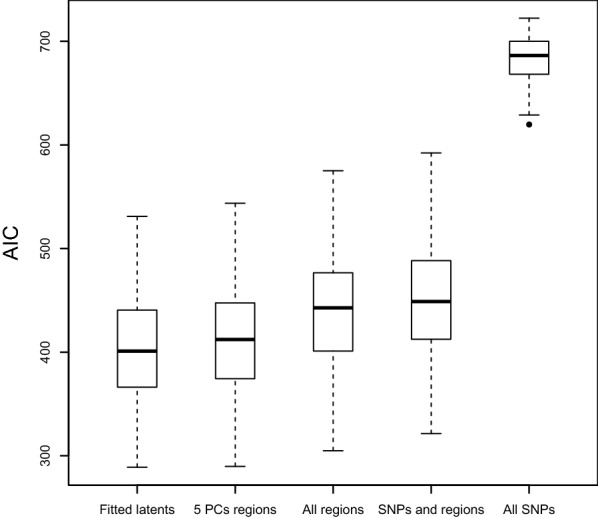
Simulation AIC model fit. The logistic regression models use either the SEM estimated latent variable scores (fitted latents), the first five principal components of the brain region data (5 PCs regions), all brain region data, all SNP and brain region data, or all SNP data

To evaluate the performance of our model, the SEM was fitted to each of the simulated data sets. We considered two measures for model comparison. First, we set out to assess the prediction of phenotypes from the latent variables, with a logistic regression. In each of the 100 simulated data sets, we estimated the latent variable scores and used only those to predict the phenotype. For each of these 100 models, we obtained an Akaike information criterion (AIC) value. We compared our model to several logistic regression models that use only the simulated data, instead of the SEM estimated latent scores. The first alternative model uses only all the brain region measurements, the second only all the SNP measurements, and the third a combination of all regions and SNP measurements. As a fourth alternative model, we performed a PCA on the volume measurements and extracted the first five principal components. Figure 2 shows that, on average, our latent variables obtain a lower AIC than models using either all brain region data, all SNP data, or both. The model using the first five principal components of the brain region data is most similar to our model, and it only has a slightly higher AIC on average than our model.

The second measure for model comparison is the ability to retrieve the correct SNPs. In each of our simulation data sets, two of the five latent variables have an effect on the phenotype (disease status). All SNPs that affect either of these two latent variables effectively impact the phenotype. We consider those SNPs to be the SNPs with a true effect. We now consider how these SNPs are ranked for importance in our SEM analysis, and two alternative approaches. From our SEM fit, we extracted the robust SNP *p* values for predicting the latent variables (so the *p* values for the estimates in ${\mathbf {A}}$). These give an impression of the importance of a SNP in predicting the latent variables. In addition, we used the latents’ logistic regression *p* values for the phenotype. These show the importance of a latent variable in predicting the phenotype. As a result, the path from a SNP to the phenotype contains two *p* values per latent variable: one for the latent variable prediction and one for the phenotype prediction.

We considered combining these *p* values in two ways: (1) for each SNP we took the maximum *p* value of the two per latent variable and then the minimum *p* value over the five latent variables; or (2) for each SNP we used Fisher’s method [26] to combine the two *p* values per latent variable ($-2 \sum \log (p_i)$) and then took the minimum *p* value over the five latent variables. Note that Fisher’s method is meant for *p* values testing the same null hypothesis, which is not the case here. Both methods yield a score (*p* value) for SNP importance. We varied a threshold for this score from 0 to 1 and compared the set of SNPs with values below this threshold to the set of SNPs with a known true effect. In this way, we constructed a receiver operating characteristic curve for SNP retrieval and calculated the corresponding area under the curve (AUC).

**Fig. 3:**
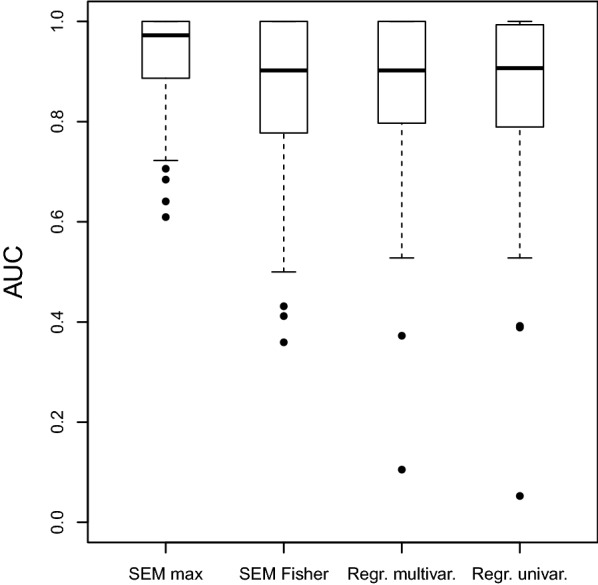
Simulation AUC for SNP selection. Shown are the results for two methods of *p* value integration for our model (SEM max and SEM Fisher), for multivariate logistic regression and univariate logistic regression. A high AUC means that the method correctly ranks the importance of the SNPs for the phenotype (disease state)

We compared the performance of our methodology to a straightforward modelling approach: a logistic regression to predict the disease status phenotype from the SNPs. This was performed both in a univariate way (as in a GWAS) and in a multivariate way. Figure 3 shows the performance of our SEM-based methods, using the maximum *p* value per SNP–latent combination (*SEM max*) or using Fisher’s method (*SEM Fisher*), and of the GWAS-like approaches. The *SEM max* method has the highest average AUC, indicating that it is best able to rank the SNPs on their importance for the phenotype. Note that the *SEM Fisher* method has the disadvantage that either a strong SNP-to-latent or a strong latent-to-phenotype effect can lead to a low combined *p* value, regardless of the other value. The observed difference between the univariate and multivariate approach is very small, which is to be expected since the simulated SNP values are independent.

To test the robustness of our model, we also compared the models for a range of alternative simulation settings. Additional file 2: Fig. S1 shows the results of these simulations. The amount of noise on the latent variables has a similar impact on all compared methods. With a large amount of noise on the brain region measurements, the prediction of phenotypes remains best with our model, but the identification of SNPs is better with methods that do not make use of these region volume data. The number of SNPs with a nonzero effect on the latent variable has little impact on the simulation results. Misspecification of the region groups, on the other hand, has a negative impact specifically on the performance of our method. This shows that our approach is somewhat sensitive to the specification of brain region groups.

### ADNI application

We apply our methodology to the Alzheimer’s Disease Neuroimaging Initiative (ADNI) data [9]. We selected 35 SNPs and 105 brain region volumes for 746 individuals. The brain regions were divided into nine region groups based on the gene expression patterns of matching brain areas in the healthy human brain [12, 21]. Each of the nine brain region groups has one corresponding latent variable, and each latent variable has a unique set of brain region measurements attached to it. Additional file 3: Fig. S2 shows the volume loadings for each of the latent variables. Since the first loading for each latent variable is set to 1, the latent variables will have the same direction of effect as this variable. All but two of the region volumes have a positive loading. Two regions in the subcortical group 2 (*SubCort2*) are negatively correlated to the latent variable scores, reflecting a more heterogeneous signal in this group.

**Fig. 4:**
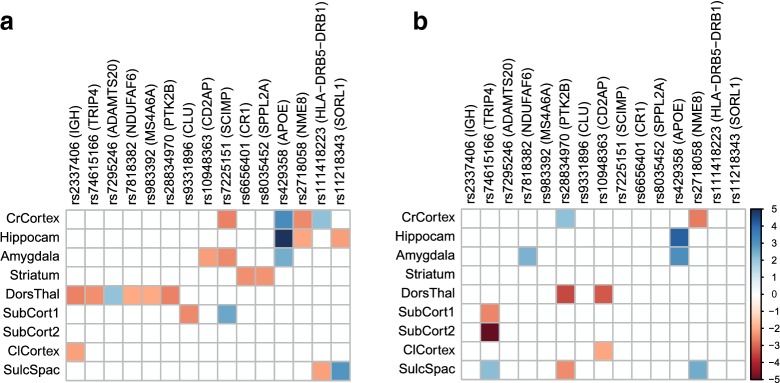
Association between SNPs and latent variable scores, as found by the robust maximum likelihood fit of the SEM. All nominally significant associations ($p<0.05$) are coloured by their robust *z*-statistic values [23]. The linked genes [25] are shown in brackets. **a** The results for the training set. After Bonferroni correction for the 315 tests, only the effect of rs429358 (*APOE*) on the hippocampus region group remains significant. **b** The validation results confirm the significant effect of rs429358 (*APOE*) on the hippocampus region group

Figure 4 shows the association between the nine latent variables and the selected SNPs. Only those SNPs are shown that have a nominally significant ($p<0.05$) association with at least one of the latent variables. After correction for multiple testing, the only significant effect is that for rs429358, located in *APOE*, on the hippocampal region group (Bonferroni-corrected $p = 2.28 \times 10^{-4}$). In the validation set, here used as a replicate, this effect was again significant (Bonferroni-corrected $p=8.66 \times 10^{-3}$). None of the other associations are significant after multiple testing correction. This *APOE* allele is known to be associated with a decrease in the hippocampal volume, both in individuals with mild cognitive impairment [27] and in Alzheimer’s disease [28].

**Fig. 5:**
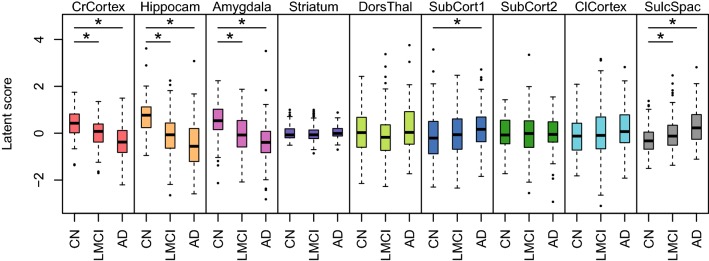
Association between the validation latent variable scores and diagnosis. Diagnosis is cognitive normal (CN), late mild cognitive impairment (LMCI), or Alzheimer’s disease (AD). Nominally significant differences (ANOVA $p<0.05$) are indicated with asterisks. The cerebral cortex (CrCortex), hippocampus (Hippocam), and amygdala (Amygdala) latent volume variables are lowered with disease progression, while the latent variable score for sulci and spaces (SulcSpac) is increased

The latent variables reflect differences in brain region volumes across the ADNI data set. To test whether these differences in brain region volumes are related to the disease phenotype, we compared the latent variable scores between the CN, LMCI, and AD individuals. Figure 5 shows the distribution of latent variable scores for the validation set. To calculate these, we used the fitted SEM of the training data and used its parameter estimates to calculate latent variable scores for the validation data. For three region groups the latent variable scores were significantly lower in LMCI than in controls, and even lower in AD. These regions are the cerebral cortex, the hippocampal formation, and the amygdala. This reflects significant shrinkage in these areas during Alzheimer’s disease progression. The region group of sulci and spaces (SulcSpac) has a latent variable that significantly increases in LMCI and AD. The significant association between the SNP rs429358 and the latent variable scores for hippocampus reflects the importance of *APOE* for Alzheimer’s disease.

## Conclusion

We have proposed the use of a maximum likelihood structural equation model for combining SNP data and structural brain area measurements. The model makes use of external gene expression data, to define groups of brain regions that may respond similarly to genetic variation. For each of these region groups, we define a latent variable, which captures the relationship between the regions in a group and genetic variation. We have applied the model to a simulated data set, to show it can capture disease-relevant variation and identify causal SNPs. In addition, we have applied the model to the ADNI data set, containing Alzheimer’s patients, individuals with late mild cognitive impairment, and cognitive healthy controls. One SNP, linked to *APOE*, shows a reproducible significant relationship to the latent variable that captures hippocampal volume change. This latent variable, and the ones representing the cerebral cortex, amygdala, and sulci and spaces, also significantly associate with the disease diagnosis. This shows that our approach can be used to integrate several data types and yield interpretable results.

The fitting process of the structural equation model has relatively high computational cost. It is truly multivariate, which makes it infeasible at the moment to perform genome-wide analysis. It does have advantages for incorporating a large number of variables, since it allows for straightforward inclusion of constraints on the parameter estimates [23]. With a constraint on the sum of squared weights, one could for instance implement a ridge regression. In addition, the model allows for the inclusion of additional data. This can be done either in the specification of the model structure, as we have done for the region groups, or by adding observed variables to the model. In our model, we chose to group brain regions based on the similarity of their expression profiles in the healthy brain. An interesting extension to the model would be to incorporate a layer of latent variables to reflect a grouping of the SNPs. These groups could also be based on the similarity of the brain-wide expression patterns of the associated genes.

These results show that maximum likelihood SEM is a versatile approach for data integration, which can be used to elucidate the relationships between genetic variation, structural brain phenotypes, and brain disease.

## Additional files

**Additional file 1: Table S1.** All brain regions used in the ADNI section, with their region group code, manually annotated ABA region, ADNI code, and ADNI description. Region group codes: CrCortex = cerebral cortex; Hippocam = hippocampal formation; Amygdala = amygdala; Striatum = striatum; DorsThal = dorsal thalamus; SubCort1 = sub-cortical regions 1; SubCort2 = sub-cortical regions 2; ClCortex = cerebellar cortex; SulcSpac = sulci and spaces.

**Additional file 2: Fig. S1.** Simulations to test model robustness. The plots show a comparison of our model (red) to alternative approaches with varying simulation parameters. We simulated a range of noise on latent variables, noise on volumes, number of SNPs, and number of misspecified latent to volume links.

**Additional file 3: Fig. S2.** Weights (loadings) from the latent variables to the region measurements. The rows correspond to latent variables (region groups), and the columns to brain regions. Each first loading per region group was set to 1, for model identifiability. The loadings show the strength of the relationship between each latent variable and the thickness/volume of its corresponding brain regions. See Additional file 1: Table S1 for the meaning of the region codes.
