# Supplementary material for: A structural equation model for imaging genetics using spatial transcriptomics
Source: Brain Inform. 2018 Nov 2;5(2):13. doi: 10.1186/s40708-018-0091-0 (PMC6429169; doi:10.1186/s40708-018-0091-0)
Supplement: Supplementary file 1 — Additional file 1: Table S1. All brain regions used in the ADNI section, with their region group code, manually annotated ABA region, ADNI code, and ADNI description. Region group codes: CrCortex = cerebral cortex; Hippocam = hippocampal formation; Amygdala = amygdala; Striatum = striatum; DorsThal = dorsal thalamus; SubCort1 = sub-cortical regions 1; SubCort2 = sub-cortical regions 2; ClCortex = cerebellar cortex; SulcSpac = sulci and spaces. [file 40708_2018_91_MOESM1_ESM.pdf]

Additional Table S1 All brain regions used in the ADNI section, with their region group code, manually annotated ABA region, ADNI code, and ADNI description. Region group codes: CrCortex = cerebral cortex; Hippocam = hippocampal formation; Amygdala = amygdala; Striatum = striatum; DorsThal = dorsal thalamus; SubCort1 = sub-cortical regions 1; SubCort2 = sub-cortical regions 2; ClCortex = cerebellar cortex; SulcSpac = sulci and spaces.

Region group code	ABA region	ADNI code	ADNI description
CrCortex	cingulate gyrus	ST14TA	Cortical Thickness Average of LeftCaudalAnteriorCingulate
CrCortex	cingulate gyrus	ST34TA	Cortical Thickness Average of LeftIsthmusCingulate
CrCortex	cingulate gyrus	ST50TA	Cortical Thickness Average of LeftPosteriorCingulate
CrCortex	cingulate gyrus	ST54TA	Cortical Thickness Average of LeftRostralAnteriorCingulate
CrCortex	cingulate gyrus	ST73TA	Cortical Thickness Average of RightCaudalAnteriorCingulate
CrCortex	cingulate gyrus	ST93TA	Cortical Thickness Average of RightIsthmusCingulate
CrCortex	cingulate gyrus	ST109TA	Cortical Thickness Average of RightPosteriorCingulate
CrCortex	cingulate gyrus	ST113TA	Cortical Thickness Average of RightRostralAnteriorCingulate
CrCortex	frontal lobe	ST25TA	Cortical Thickness Average of LeftFrontalPole
CrCortex	frontal lobe	ST36TA	Cortical Thickness Average of LeftLateralOrbitofrontal
CrCortex	frontal lobe	ST39TA	Cortical Thickness Average of LeftMedialOrbitofrontal
CrCortex	frontal lobe	ST43TA	Cortical Thickness Average of LeftParacentral
CrCortex	frontal lobe	ST45TA	Cortical Thickness Average of LeftParsOpercularis
CrCortex	frontal lobe	ST46TA	Cortical Thickness Average of LeftParsOrbitalis
CrCortex	frontal lobe	ST47TA	Cortical Thickness Average of LeftParsTriangularis
CrCortex	frontal lobe	ST51TA	Cortical Thickness Average of LeftPrecentral
CrCortex	frontal lobe	ST55TA	Cortical Thickness Average of LeftRostralMiddleFrontal
CrCortex	frontal lobe	ST56TA	Cortical Thickness Average of LeftSuperiorFrontal
CrCortex	frontal lobe	ST84TA	Cortical Thickness Average of RightFrontalPole
CrCortex	frontal lobe	ST95TA	Cortical Thickness Average of RightLateralOrbitofrontal
CrCortex	frontal lobe	ST98TA	Cortical Thickness Average of RightMedialOrbitofrontal
CrCortex	frontal lobe	ST102TA	Cortical Thickness Average of RightParacentral
CrCortex	frontal lobe	ST104TA	Cortical Thickness Average of RightParsOpercularis
CrCortex	frontal lobe	ST105TA	Cortical Thickness Average of RightParsOrbitalis
CrCortex	frontal lobe	ST106TA	Cortical Thickness Average of RightParsTriangularis
CrCortex	frontal lobe	ST110TA	Cortical Thickness Average of RightPrecentral
CrCortex	frontal lobe	ST114TA	Cortical Thickness Average of RightRostralMiddleFrontal
CrCortex	frontal lobe	ST115TA	Cortical Thickness Average of RightSuperiorFrontal
CrCortex	insula	ST129TA	Cortical Thickness Average of LeftInsula
CrCortex	insula	ST130TA	Cortical Thickness Average of RightInsula
CrCortex	middle frontal gyrus	ST15TA	Cortical Thickness Average of LeftCaudalMiddleFrontal
CrCortex	middle frontal gyrus	ST74TA	Cortical Thickness Average of RightCaudalMiddleFrontal
CrCortex	occipital lobe	ST23TA	Cortical Thickness Average of LeftCuneus
CrCortex	occipital lobe	ST35TA	Cortical Thickness Average of LeftLateralOccipital
CrCortex	occipital lobe	ST38TA	Cortical Thickness Average of LeftLingual
CrCortex	occipital lobe	ST48TA	Cortical Thickness Average of LeftPericalcarine
CrCortex	occipital lobe	ST82TA	Cortical Thickness Average of RightCuneus
CrCortex	occipital lobe	ST94TA	Cortical Thickness Average of RightLateralOccipital
CrCortex	occipital lobe	ST97TA	Cortical Thickness Average of RightLingual
CrCortex	occipital lobe	ST107TA	Cortical Thickness Average of RightPericalcarine
CrCortex	parahippocampal gyrus	ST44TA	Cortical Thickness Average of LeftParahippocampal
CrCortex	parahippocampal gyrus	ST103TA	Cortical Thickness Average of RightParahippocampal
CrCortex	parietal lobe	ST31TA	Cortical Thickness Average of LeftInferiorParietal
CrCortex	parietal lobe	ST49TA	Cortical Thickness Average of LeftPostcentral
CrCortex	parietal lobe	ST52TA	Cortical Thickness Average of LeftPrecuneus
CrCortex	parietal lobe	ST57TA	Cortical Thickness Average of LeftSuperiorParietal
CrCortex	parietal lobe	ST59TA	Cortical Thickness Average of LeftSupramarginal
CrCortex	parietal lobe	ST90TA	Cortical Thickness Average of RightInferiorParietal
CrCortex	parietal lobe	ST108TA	Cortical Thickness Average of RightPostcentral
CrCortex	parietal lobe	ST111TA	Cortical Thickness Average of RightPrecuneus
CrCortex	parietal lobe	ST116TA	Cortical Thickness Average of RightSuperiorParietal
CrCortex	parietal lobe	ST118TA	Cortical Thickness Average of RightSupramarginal
CrCortex	temporal lobe	ST13TA	Cortical Thickness Average of LeftBankssts
CrCortex	temporal lobe	ST24TA	Cortical Thickness Average of LeftEntorhinal
CrCortex	temporal lobe	ST26TA	Cortical Thickness Average of LeftFusiform
CrCortex	temporal lobe	ST32TA	Cortical Thickness Average of LeftInferiorTemporal
CrCortex	temporal lobe	ST40TA	Cortical Thickness Average of LeftMiddleTemporal
CrCortex	temporal lobe	ST58TA	Cortical Thickness Average of LeftSuperiorTemporal
CrCortex	temporal lobe	ST60TA	Cortical Thickness Average of LeftTemporalPole
CrCortex	temporal lobe	ST62TA	Cortical Thickness Average of LeftTransverseTemporal
CrCortex	temporal lobe	ST72TA	Cortical Thickness Average of RightBankssts
CrCortex	temporal lobe	ST83TA	Cortical Thickness Average of RightEntorhinal
CrCortex	temporal lobe	ST85TA	Cortical Thickness Average of RightFusiform
CrCortex	temporal lobe	ST91TA	Cortical Thickness Average of RightInferiorTemporal
CrCortex	temporal lobe	ST99TA	Cortical Thickness Average of RightMiddleTemporal
CrCortex	temporal lobe	ST117TA	Cortical Thickness Average of RightSuperiorTemporal
CrCortex	temporal lobe	ST119TA	Cortical Thickness Average of RightTemporalPole
CrCortex	temporal lobe	ST121TA	Cortical Thickness Average of RightTransverseTemporal
Hippocam	hippocampal formation	ST29SV	Volume (WM Parcellation) of LeftHippocampus
Hippocam	hippocampal formation	ST88SV	Volume (WM Parcellation) of RightHippocampus
Amygdala	amygdala	ST12SV	Volume (WM Parcellation) of LeftAmygdala
Amygdala	amygdala	ST71SV	Volume (WM Parcellation) of RightAmygdala
Striatum	striatum	ST11SV	Volume (WM Parcellation) of LeftAccumbensArea
Striatum	striatum	ST16SV	Volume (WM Parcellation) of LeftCaudate
Striatum	striatum	ST53SV	Volume (WM Parcellation) of LeftPutamen
Striatum	striatum	ST70SV	Volume (WM Parcellation) of RightAccumbensArea
Striatum	striatum	ST75SV	Volume (WM Parcellation) of RightCaudate

Additional Table S1: Continued

Striatum	striatum	ST112SV	Volume (WM Parcellation) of RightPutamen
DorsThal	dorsal thalamus	ST61SV	Volume (WM Parcellation) of LeftThalamus
DorsThal	dorsal thalamus	ST120SV	Volume (WM Parcellation) of RightThalamus
SubCort1	myelencephalon	ST1SV	Volume (WM Parcellation) of Brainstem
SubCort2	globus pallidus	ST42SV	Volume (WM Parcellation) of LeftPallidum
SubCort2	globus pallidus	ST101SV	Volume (WM Parcellation) of RightPallidum
SubCort2	white matter	ST2SV	Volume (WM Parcellation) of CorpusCallosumAnterior
SubCort2	white matter	ST3SV	Volume (WM Parcellation) of CorpusCallosumCentral
SubCort2	white matter	ST4SV	Volume (WM Parcellation) of CorpusCallosumMidAnterior
SubCort2	white matter	ST5SV	Volume (WM Parcellation) of CorpusCallosumMidPosterior
SubCort2	white matter	ST6SV	Volume (WM Parcellation) of CorpusCallosumPosterior
SubCort2	white matter	ST18SV	Volume (WM Parcellation) of LeftCerebellumWM
SubCort2	white matter	ST20SV	Volume (WM Parcellation) of LeftCerebralWM
SubCort2	white matter	ST69SV	Volume (WM Parcellation) of OpticChiasm
SubCort2	white matter	ST77SV	Volume (WM Parcellation) of RightCerebellumWM
SubCort2	white matter	ST79SV	Volume (WM Parcellation) of RightCerebralWM
SubCort2	white matter	ST128SV	Volume (WM Parcellation) of WMHypoIntensities
ClCortex	cerebellar cortex	ST17SV	Volume (WM Parcellation) of LeftCerebellumCortex
ClCortex	cerebellar cortex	ST76SV	Volume (WM Parcellation) of RightCerebellumCortex
SulcSpac	sulci & spaces	ST7SV	Volume (WM Parcellation) of Csf
SulcSpac	sulci & spaces	ST9SV	Volume (WM Parcellation) of FourthVentricle
SulcSpac	sulci & spaces	ST21SV	Volume (WM Parcellation) of LeftChoroidPlexus
SulcSpac	sulci & spaces	ST30SV	Volume (WM Parcellation) of LeftInferiorLateralVentricle
SulcSpac	sulci & spaces	ST37SV	Volume (WM Parcellation) of LeftLateralVentricle
SulcSpac	sulci & spaces	ST80SV	Volume (WM Parcellation) of RightChoroidPlexus
SulcSpac	sulci & spaces	ST89SV	Volume (WM Parcellation) of RightInferiorLateralVentricle
SulcSpac	sulci & spaces	ST96SV	Volume (WM Parcellation) of RightLateralVentricle
SulcSpac	sulci & spaces	ST127SV	Volume (WM Parcellation) of ThirdVentricle
