# Supplementary material for: A structural equation model for imaging genetics using spatial transcriptomics
Source: Brain Inform. 2018 Nov 2;5(2):13. doi: 10.1186/s40708-018-0091-0 (PMC6429169; doi:10.1186/s40708-018-0091-0)
Supplement: Supplementary file 2 — Additional file 2: Fig. S1. Simulations to test model robustness. The plots show a comparison of our model (red) to alternative approaches with varying simulation parameters. We simulated a range of noise on latent variables, noise on volumes, number of SNPs, and number of misspecified latent to volume links. [file 40708_2018_91_MOESM2_ESM.pdf]

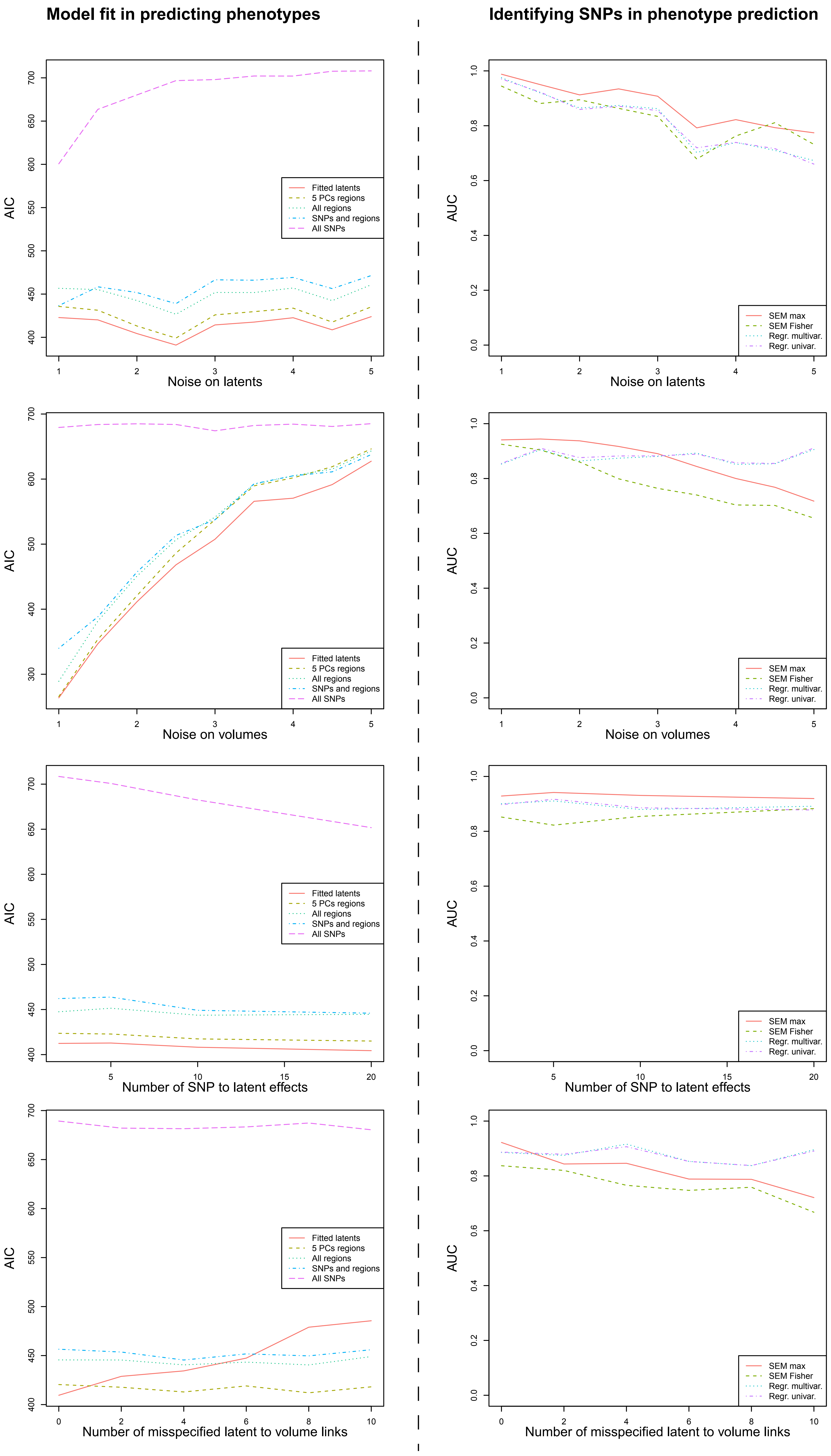

Additional Fig. S1 Simulations to test model robustness. The plots show a comparison of our model (red) to alternative approaches with varying simulation parameters. We simulated a range of noise on latent variables, noise on volumes, number of SNPs, and number of misspecified latent to volume links.
