# Supplementary material for: A structural equation model for imaging genetics using spatial transcriptomics
Source: Brain Inform. 2018 Nov 2;5(2):13. doi: 10.1186/s40708-018-0091-0 (PMC6429169; doi:10.1186/s40708-018-0091-0)
Supplement: Supplementary file 3 — Additional file 3: Fig. S2. Weights (loadings) from the latent variables to the region measurements. The rows correspond to latent variables (region groups), and the columns to brain regions. Each first loading per region group was set to 1, for model identifiability. The loadings show the strength of the relationship between each latent variable and the thickness/volume of its corresponding brain regions. See Additional file 1: Table S1 for the meaning of the region codes. [file 40708_2018_91_MOESM3_ESM.pdf]

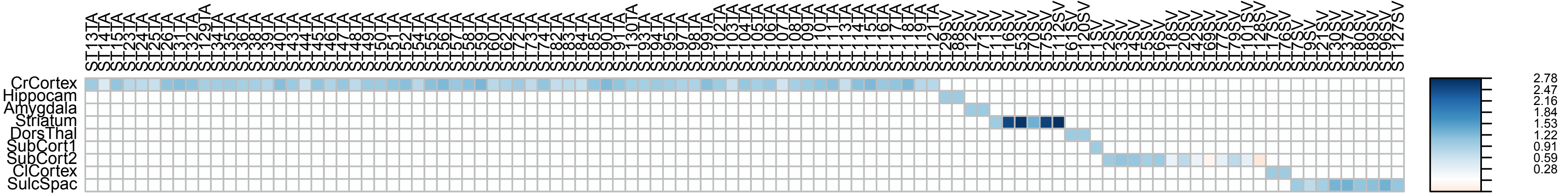

Additional Fig. S2 Weights (loadings) from the latent variables to the region measurements. The rows correspond to latent variables (region groups), and the columns to brain regions. Each first loading per region group was set to 1, for model identifiability. The loadings show the strength of the relationship between each latent variable and the thickness/volume of its corresponding brain regions. See Additional Table S1 for the meaning of the region codes.
